# Stereotactically-navigated percutaneous Irreversible Electroporation (IRE) compared to conventional IRE: a prospective trial

**DOI:** 10.7717/peerj.2277

**Published:** 2016-08-11

**Authors:** Lukas P. Beyer, Benedikt Pregler, Christoph Nießen, Andreas Schicho, Michael Haimerl, Ernst Michael Jung, Christian Stroszczynski, Philipp Wiggermann

**Affiliations:** Department of Radiology, University Hospital Regensburg, Regensburg, Germany

**Keywords:** Interventional radiology, Robotic assistance, Irreversible electroporation, Liver tumor, CT-guided, Stereotactic navigation

## Abstract

**Purpose.** The purpose of this study was to compare CT-navigated stereotactic IRE (SIRE) needle placement to non-navigated conventional IRE (CIRE) for percutaneous ablation of liver malignancies.

**Materials and Methods.** A prospective trial including a total of 20 patients was conducted with 10 patients in each arm of the study. IRE procedures were guided using either CT fluoroscopy (CIRE) or a stereotactic planning and navigation system (SIRE). Primary endpoint was procedure time. Secondary endpoints were accuracy of needle placement, technical success rate, complication rate and dose-length product (DLP).

**Results.** A total of 20 IRE procedures were performed to ablate hepatic malignancies (16 HCC, 4 liver metastases), 10 procedures in each arm. Mean time for placement of IRE electrodes in SIRE was significantly shorter with 27 ± 8 min compared to 87 ± 30 min for CIRE (*p* < 0.001). Accuracy of needle placement for SIRE was higher than CIRE (2.2 mm vs. 3.3 mm mean deviation, *p* < 0.001). The total DLP and the fluoroscopy DLP were significantly lower in SIRE compared to CIRE. Technical success rate and complication rates were equal in both arms.

**Conclusion.** SIRE demonstrated a significant reduction of procedure length and higher accuracy compared to CIRE. Stereotactic navigation has the potential to reduce radiation dose for the patient and the radiologist without increasing the risk of complications or impaired technical success compared to CIRE.

## Introduction

Irreversible Electroporation (IRE) is a novel method for focused treatment of liver tumors (Rubinsky, 2007). IRE is a soft tissue ablation technique using ultra-short but strong electrical fields to create permanent and hence lethal nanopores in the cell membrane in order to disrupt cellular homeostasis (Davalos, Mir & Rubinsky, 2005). The cell death results mainly from apoptosis and not necrosis as in all other thermal or radiation-based ablation techniques although local coagulation necrosis has been shown in the immediate proximity to the electrodes (Ben-David et al., 2013). IRE is used for non-resectable liver tumors in the vicinity of vessels (due to its selectivity for tumor tissue while preserving vessel structures as well as the absence of the so-called heat sink effect) (Rubinsky, Onik & Mikus, 2007). IRE has also recently shown therapeutic efficacy and safety in other organs like pancreas and prostate (Martin et al., 2015; Ting et al., 2016). First animal studies have shown the safety and feasibility of stereotactically delivered IRE for the treatment of telencephalic gliomas (Rossmeisl et al., 2015).

IRE ablation requires the placement of two or more applicator electrodes between which the electrical fields are applied. In order to achieve successful ablation, parallel needle placement at a pre-defined distance between 1.5 cm and 2 cm is required. Mathematic models have shown that parallel placement of IRE electrodes is essential to generate an even distribution of the electromagnetic field (Edd & Davalos, 2007). This fact was also shown in porcine animal model by Ben-David et al. (2013) who investigated the therapeutic efficacy of IRE with regard to electrode orientation, tissue type and local environment.

Needles are placed under image guidance using ultrasound or computer tomography as imaging methods. Since these methods display one image plane at a time, the realization of multiple parallel needle placements can be challenging. Several attempts may be necessary to achieve the required geometrical configuration of the needle with respect to other needles and as well as in relation to the anatomical target.

Navigation technology for interventional radiology supports IRE treatments by providing comprehensive planning of needle configurations using 3D image data and by supporting needle placement through guidance functionality.

This study aims to investigate the potential benefits of CT-navigated stereotactic IRE (SIRE) needle placement compared to non-navigated conventional IRE (CIRE) for ablation of malignant liver lesions.

## Materials and Methods

### Study design, participant selection and patient characteristics

In a prospective, non-blinded, non-randomized two-armed study carried out between July 2015 and February 2016, IRE ablations of malignant liver tumors were performed in 10 procedures with stereotactic navigation and 10 cases without. The primary end point of the study was the time required until start of the ablation (measured from the time of the first CT scan to the start of the ablation). Secondary endpoints included accuracy of IRE electrode placement (accuracy is measured as lateral deviation of the IRE electrodes to a central reference electrode); overall procedure time; number of needle replacements; radiation dose and number of control scans.

The study has been approved by the Ethics Committee of the University Regensburg (approval number 15-101-0188) and written consent was obtained from all patients. All procedures were performed according to the Declaration of Helsinki and the guideline for Good Clinical Practice from the International Conference on Harmonization.

In all cases, indication for percutaneous tumor ablation was determined by an interdisciplinary tumor board. An IRE was indicated if surgical resection was not possible, i.e., because no R0 resection was possible or an increased risk of insufficient hepatic functional reserve, and if radiofrequency ablation (RFA) and microwave ablation (MWA) were contraindicated. Exclusion criteria included any condition which, in the judgment of the clinical investigator or his designee, might increase the risk to the subject or decrease the chance of obtaining satisfactory data to achieve the objectives of the study. Patients with hereditary hematological / coagulation disorders unrelated to their liver disease were likewise excluded. Patients who were currently (within the last 30 days prior to surgery) participating in another clinical trial with any investigational drug or device were ruled out, as well as those patients undergoing liver surgery for the purpose of receiving a liver transplant.

**Figure 1:**
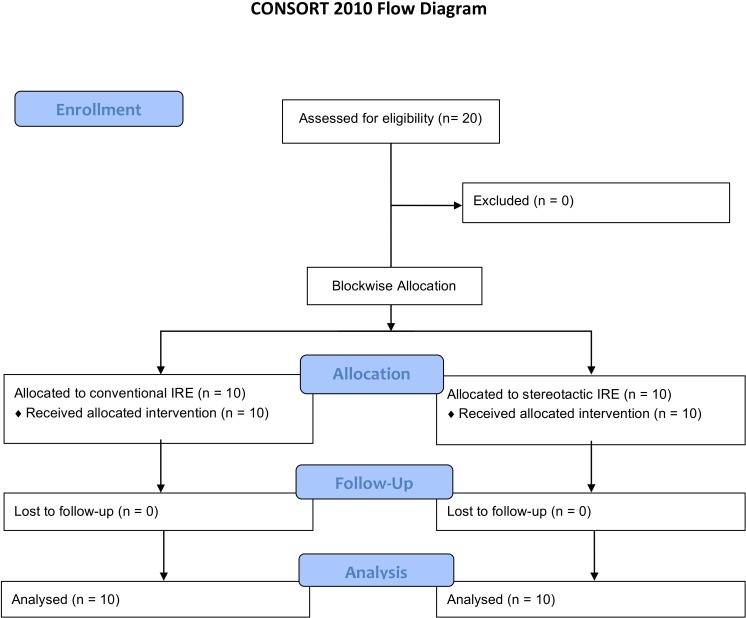
Flow of participants through each stage of the study.

The registration of the study (ISRCTN55383115) was applied after it was completed, since the project was initially conducted as an internal evaluation of the navigation device. In total the study comprised 20 IRE procedures performed on primary liver tumors and liver metastases in 20 patients (two female, 18 male, average age 66 years, age range 46–81 years). The first 10 procedures were performed using CT fluoroscopy without navigation assistance (CIRE), the other 10 using stereotactic navigation (SIRE; Fig. 1).

In all cases a pre-interventional MRI examination had been performed using liver-specific contrast (Primovist, Bayer Schering Pharma, Berlin) as reference imaging. 16 of the 20 ablated lesions were hepatocellular carcinomas; the other four were colorectal liver metastases (Table 1).

**Table 1:** Number of ablated lesions with and without stereotactic navigation.

Conventional or stereotactic	SIRE	CIRE
Hepatocellular carcinoma	10	6
Metastasis of colorectal cancer	0	4
All	10	10

### IRE procedure

All interventions were performed under full anesthesia with deep muscle relaxation by one experienced interventional radiologist (more than 400 percutaneous tumor ablations done). After acquisition of an arterial and portal venous planning CT (Somatom Sensation 16; Siemens Healthcare, Forchheim, Germany) with respiration control (endotracheal tube disconnection), DICOM data were transferred optionally to the navigation system (CAS-One I; CAScination AG, Bern, Switzerland) or to manual planning on the PACS system.

In the SIRE group radiopaque optical markers were placed on the patient before acquisition of the planning CT. The navigation system was placed next to the CT gantry and the optical tracking camera was set up above the patient in a way, that it could effectively track both the optical markers placed on the patient and the navigation device.

Programming of the IRE generator (NanoKnife System; AngioDynamics^®^, Latham, New York, NY, USA) was performed in both arms of the study following the manufacturer’s instructions: electric field, 1,500 V/cm needle distance; pulse length, 90 µs; pulses per cycle, 90. Before delivering the 90 therapeutic pulses, a test pulse at 270 V was delivered. After the test pulse confirmed adequate conductivity, 90 pulses were delivered in less than 2 min. When the current generated by the electrodes exceeded 48 amps, those electrodes were withdrawn from the therapeutic algorithm and pulses between those electrodes were aborted.

Post-interventionally all patients received 20 mg Enoxaparin subcutaneously once a day until full mobilization.

### Non-navigated conventional IRE (CIRE)

CT fluoroscopy (CARE Vision, Somatom Sensation 16; Siemens Healthcare, Forchheim, Germany; CT parameters during fluoroscopy: tube voltage 120 kVp; effective tube current-time product 30 mAs; slice collimation 16 mm × 0.75 mm) is an acquisition mode that allows continuous image update using in-room table control. A final position control scan was performed after iterative insertion of 2–6 monopolar 18-gauge ablation electrodes by needle advancement and using image control.

### Navigated stereotactic IRE (SIRE)

Prior to acquisition of the planning CT, the patient was immobilized in a vacuum fixation system (iSYS Medizintechnik GmbH, Kitzbühl, Austria), and optical fiducial markers were attached to the patients’ chests to register image-to-patient coordinates. Based on the transmitted DICOM data, the navigation system software was used to define the tumor localization as well as to plan the access path of the IRE electrodes.

**Figure 2:**
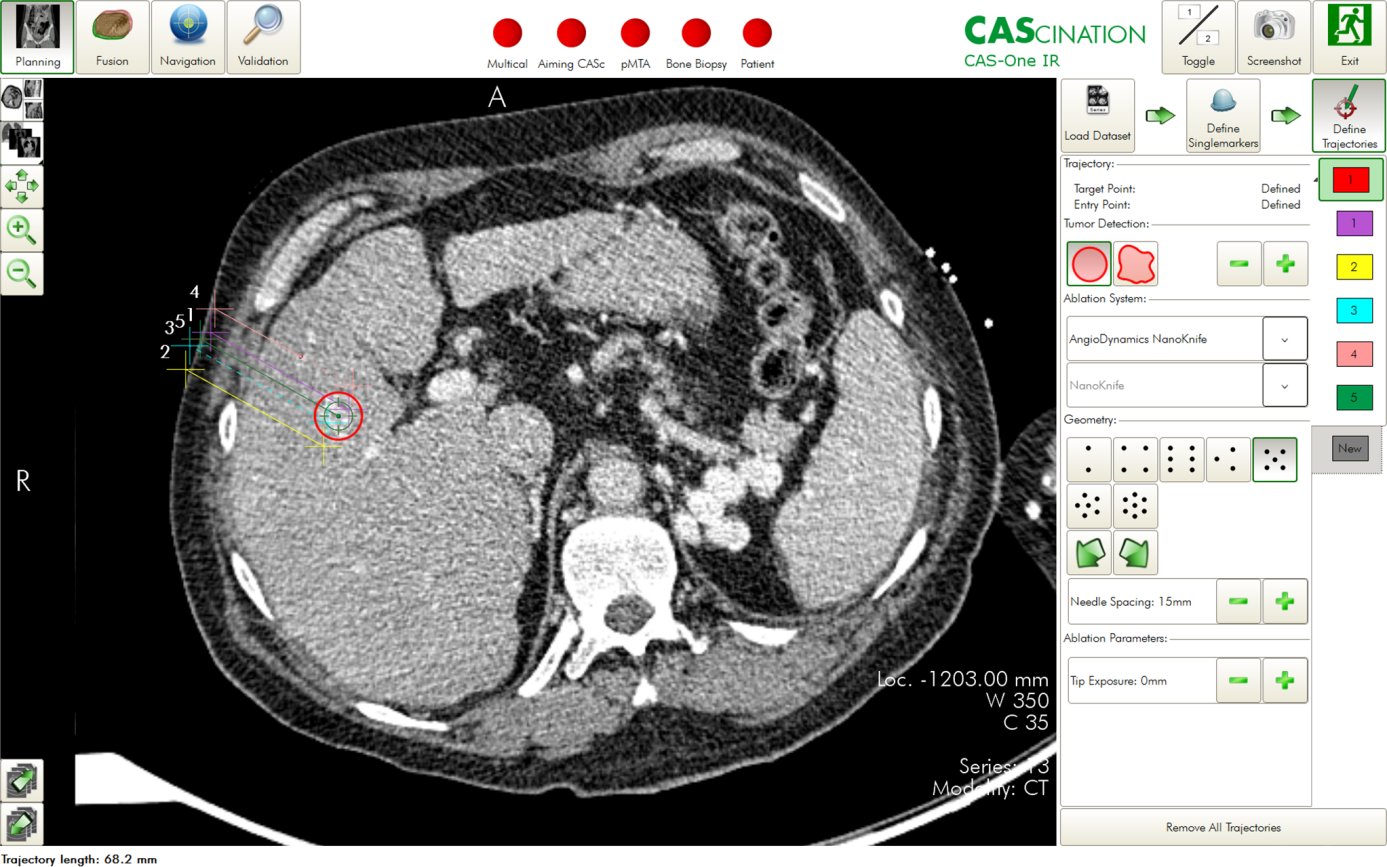
Planning the path of access on the navigation system. A virtual entry point on the skin and tumor center is defined. Then the trajectories of the individual electrodes are calculated, but can still be adjusted individually.

For parallel electrode placement, the software allows the selection of different geometric patterns depending on the number of required electrodes, e.g., rectangular or three-cornered shapes (Fig. 2). The entry point on the skin and distance between the electrodes have to be determined by the interventionalist. Each trajectory can be individually adjusted to avoid critical structures.

**Figure 3:**
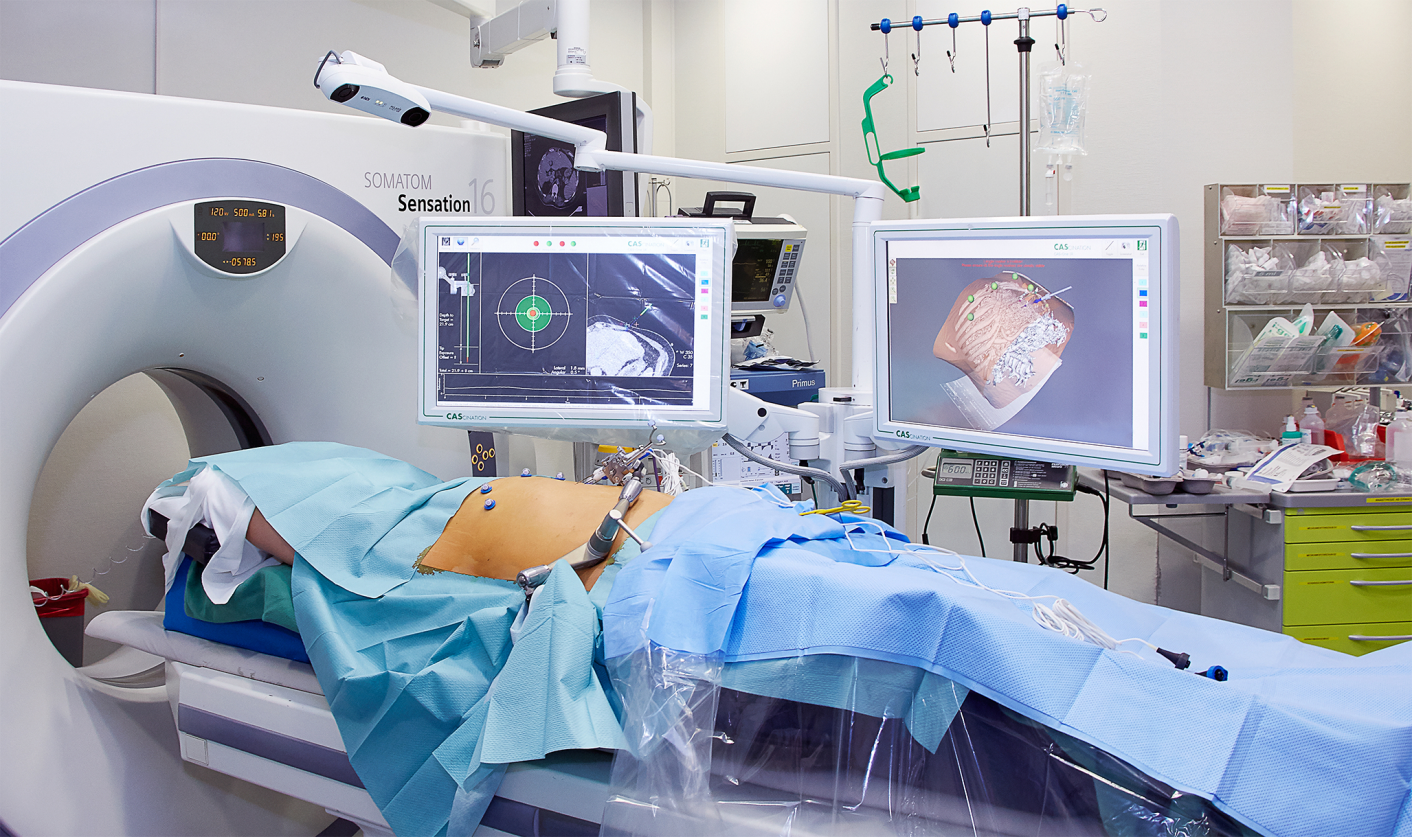
Positioning the needle guidance device for inserting the IRE electrodes.

Upon the conclusion of planning, the needle guidance device was aligned with the navigation system to the planned path of access, and the electrode was inserted (Fig. 3). Once all electrodes were placed, a needle position control scan was performed.

### Radiation exposure dose

The total dose-length product (DLP), fluoroscopy DLP and the number of verification scans to check the location of the needle during the intervention were recorded.

### Procedural accuracy

**Figure 4:**
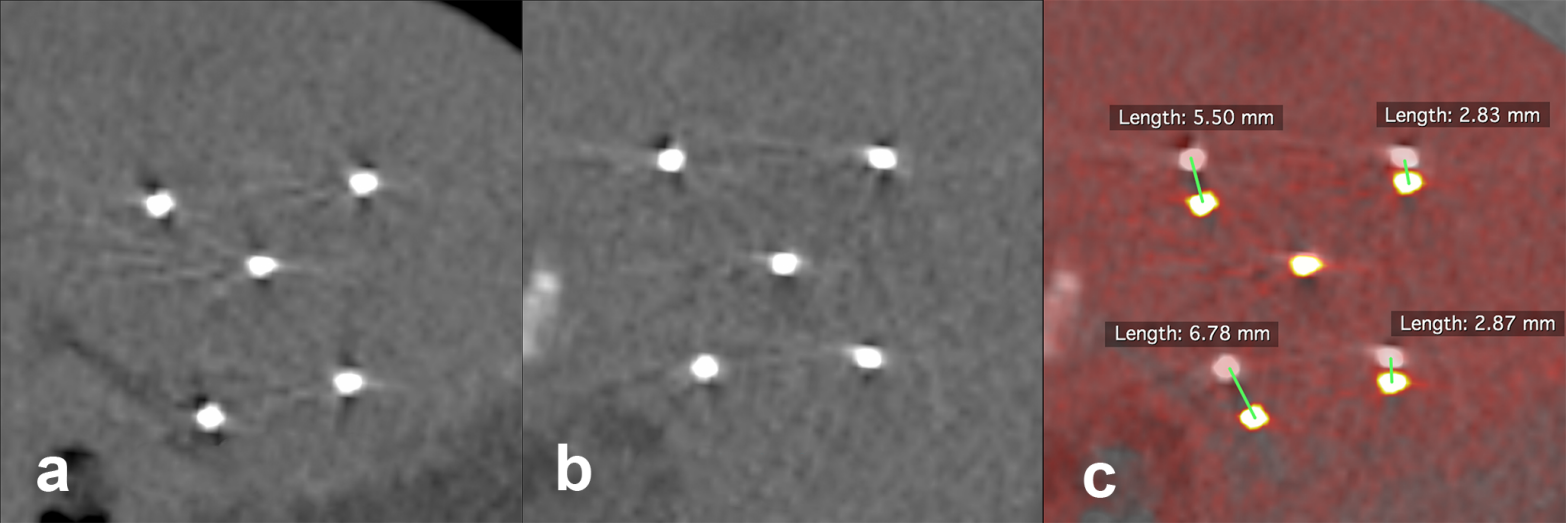
Evaluation of the lateral deviation of the IRE electrodes. (A) Orthogonal plane (thickness 0.7 mm) at the tip of the reference electrode. (B) Orthogonal plane at a distance of 3 cm from the tip of the reference electrode. (C) Fusion of (A) and (B) with determination of lateral probe deviations.

Accuracy of IRE needle placement is measured as the degree of parallelism defined as the lateral deviation of each IRE electrode over the last 3 cm (from the probe tip) with respect to a reference electrode defined as the probe in the most central position in the tumor (Fig. 4).

### Complications

Complications were documented and classified as minor and major complications according to the standardized grading system of the Society of Interventional Radiology (Omary et al., 2003).

### Follow-up

**Figure 5:**
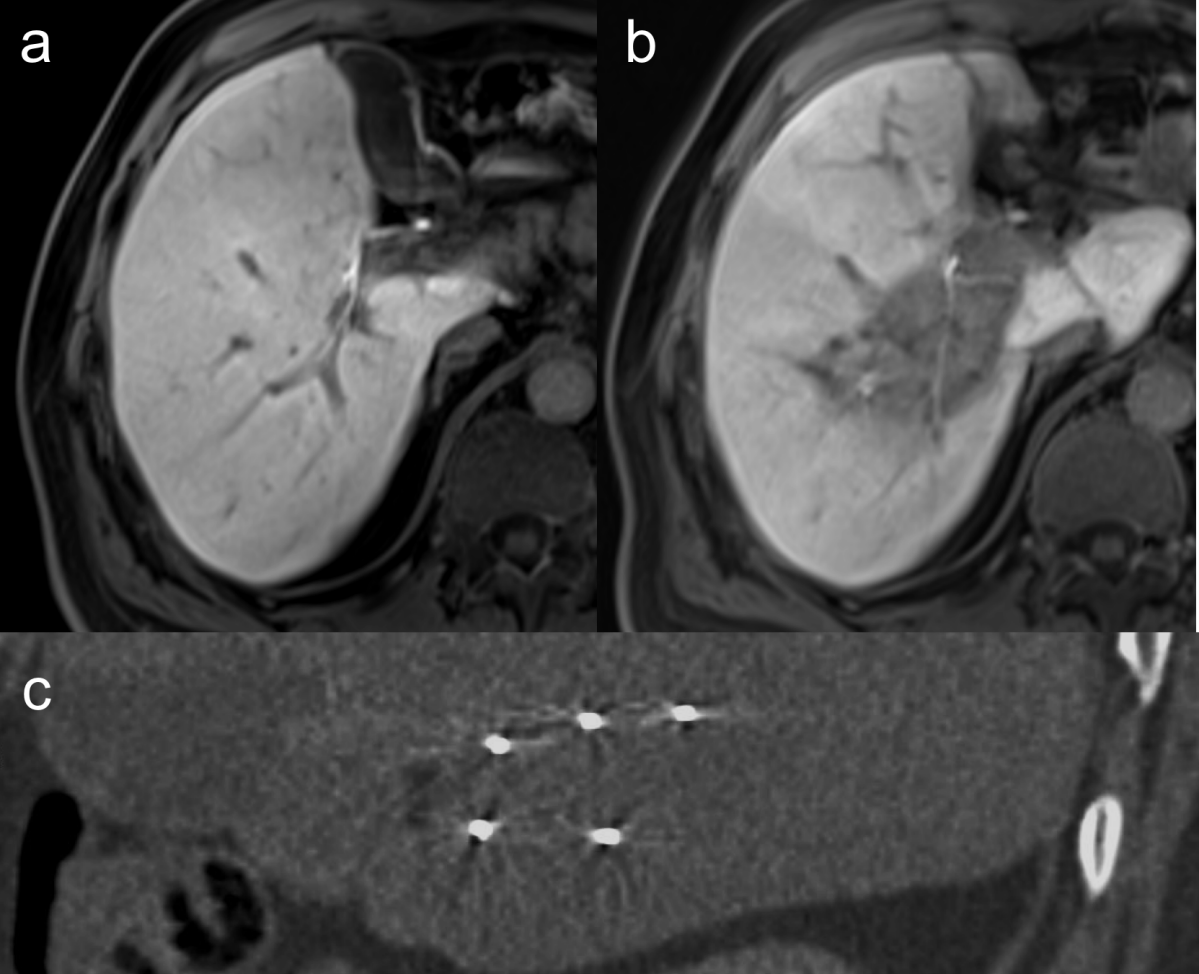
Colorectal liver metastasis in close relationship to the right portal vein. (A) Late phase MRI shows complete ablation (B) after stereotactic placement of 3 IRE electrodes above and 2 below the portal vein (C).

All patients underwent a 6-week follow-up including an MRI with liver-specific contrast agent of the liver to assess complete ablation, i.e., technical success of the ablation procedure (Fig. 5). Ablations were considered complete when no areas of enhancement were seen in the tumor or at the periphery. Images were analyzed by two experienced radiologists in consensus reading.

### Statistical analysis

The statistical software packages JMP (SAS Institute, Cary, NC, USA) and R (R Core Team, 2015) were used to perform the statistical analysis. Data were expressed as mean ± standard deviation. Outcomes were compared using Welch’s *t*-test for unequal variances with the exception of electrode deviation, which was compared using the *t*-test for clustered data. *T*-test statistics are reported with degrees of freedom in parentheses, the *t* statistic and the significance level. A *p*-value of *p* ≤ 0.05 was considered the cut-off point of statistical significance.

## Results

### Tumor characteristics

Baseline patient characteristics and lesion aspects are summarized in Table 2.

**Table 2:** Base patient and lesion aspects. Lesion conspicuity denotes the difference in attenuation between tumor and liver parenchyma in Hounsfield units (*HU*_*liver*_ − *HU*_*lesion*_). Data are presented as means and standard deviations.

Conventional or stereotactic	CIRE (*n* = 10)	SIRE (*n* = 10)
Sex (male)	9 (90 %)	9 (90 %)
Age—years	63.2 ± 8.6	69.0 ± 11.9
BMI—kg/m^2^	28.4 ± 4.1	29.0 ± 3.1
Skin to tumor depth—mm	79.8 ± 26.9	67.7 ± 25.2
Tumor long axis—mm	25.4 ± 8.1	25.0 ± 9.0
Tumor conspicuity native—HU	10.6 ± 7.8	12.8 ± 5.6
Tumor conspicuity enhanced—HU	35.6 ± 14.2	29.1 ± 7.6

### Procedural accuracy

The average deviation of the IRE electrodes with respect to the reference electrode was 3.3 mm ± 1.2 (range 0.8 mm–6.2 mm) for CIRE and 2.2 mm ± 0.9 (range 0.6 mm–4.0 mm) for SIRE with the difference being statistically significant (*p* < 0.001). The corresponding deviation expressed in degrees of arc was 9.3 ± 3.3 vs. 6.4 ± 2.6 (CIRE vs. SIRE).

### Procedural duration

The average time required to complete sterile patient preparation, the electrode placement time and the procedure time are reported in Table 3. In contrast to CIRE, SIRE requires definition of the trajectories in the navigation system which required 12.3 min ± 4.0.

**Table 3:** Time required for sterile patient preparation; placement of IRE electrodes and total intervention. Data are presented as means and standard deviations.

Conventional or stereotactic	CIRE (*n* = 10)	SIRE (*n* = 10)	*t*-test
Sterile patient preparation duration—min	17.1 ± 2.8	16.1 ± 3.8	*t*(16.4) = 0.67, *p* = 0.514
Electrode placement duration—min	87.0 ± 29.9	26.8 ± 7.7	*t*(10.2) = 6.2, *p* < 0.001
Placement time per electrode—min	18.0 ± 4.2	5.9 ± 2.0	*t*(13.0) = 8.1, *p* < 0.001
Procedure time until start of the ablation—min	104.1 ± 28.2	55.2 ± 9.3	*t*(10.9) = 5.2, *p* < 0.001

### Radiation dose

The DLP of the entire intervention, the CT fluoroscopy and the verification scans were significantly lower in SIRE compared to CIRE (Table 4).

**Table 4:** Dose length product (DLP) of the entire intervention, CT fluoroscopy and all verification scans. Data are presented as means and standard deviations.

Conventional or stereotactic	CIRE (*N* = 10)	SIRE (*N* = 10)	*t*-test
Total DLP—mGy*cm	4886 ± 1775	3510 ± 887	*t*(13.2)=2.2, *p* = 0.047
Verification DLP—mGy*cm	1351 ± 479	725 ± 326	*t*(15.8) = 3.4, *p* = 0.004
Fluoroscopy DLP—mGy*cm	1705 ± 1583	136 ± 206	*t*(9.3) = 3.1, *p* = 0.012

### Ablation success

In the follow-up after six weeks, complete ablation without residual tumor was seen in 100% (10 of 10) of robot-assisted ablation cases and in 100% (10 of 10) of manual ablation cases.

### Complications

There were no complications in navigation-assisted and manual ablation.

## Discussion

The aim of the present study was to evaluate stereotactic percutaneous IRE of malignant liver tumors for the first time as a promising alternative to conventional percutaneous IRE using CT fluoroscopy.

Both patient groups were comparable with regards to baseline patient and tumor characteristics (Table 2). The difference of 12 mm in skin to tumor depth between both groups was neither clinically nor statistically significant.

IRE relies on increasing the permeability of the cell membrane based on nanopores induced by a strong electromagnetic field (Davalos, Mir & Rubinsky, 2005). If the electrical field is powerful enough, the cell membrane is sufficiently damaged so that its integrity is permanently altered, allowing the induction of apoptosis in the cell (Yarmush et al., 2014; Jiang, Davalos & Bischof, 2015). Although new bipolar IRE electrodes are being developed only monopolar electrodes are currently commercially available. These monopolar electrodes must be placed parallel in order to generate a sufficiently strong electromagnetic field within the entire tumor or ablation zone (Martin, 2013; Scheffer et al., 2015). The consequent complexity of probe placement results in significantly longer intervention times compared to thermal interventional methods. Thus, average intervention times of 86 min have been reported for RFA of liver tumors supported by CT fluoroscopy (Basdanis et al., 2004). On the other hand, intervention took an average of 170 min for percutaneous CIRE (Ball, Thomson & Kavnoudias, 2010), primary due to the time-consuming manual placement of the individual probes using CT fluoroscopy.

Deviations detected in the course of control scans and resulting repositioning of individual probes represent a significant component of the distinctly longer intervention times for IRE when compared to thermal ablation. In this context, the evaluation of the benefit of stereotactic navigation technology for interventional radiology during IRE procedures was the primary aim of this study. It was demonstrated that 3D treatment planning on the navigation equipment resulted in an average planning time of 12.3 min. However, in the end, electrode placement in the course of SIRE was distinctly faster with 5.9 min per electrode, compared to 18.0 min during CIRE. This likewise resulted in a significant decrease in the total intervention time until ablation (104 min vs. 55 min; *p* < 0.001).

In the course of a prospective study of 70 patients, Mbalisike et al. (2014) determined that robot-supported percutaneous microwave ablation provides very high precision. According to their measurements there was a minor average deviation of the active center of the microwave probe (1.9 mm) compared to the center of the tumor. Our own studies using robot-assisted MWA of a total of 64 liver tumors found a comparably minor deviation of 1.3 mm (Beyer et al., 2015).

The distance of the electrodes from the tumor center is not a suitable measure for determining the accuracy of the placement of the IRE electrodes, since they have to be inserted in the periphery of the tumor as well as in the tumor center. Unlike thermal ablation procedures, exactly parallel insertion of the IRE probes into the liver tissue is decisive for therapeutic success. Therefore, in the course of our study, parallelism of the inserted electrodes was evaluated as such rather than their relation to the tumor center. As a result, for SIRE our study indicated an average deviation of 2.2 mm compared to 3.3 mm for insertion of electrodes using CT fluoroscopy.

Systematic errors, i.e., deviation of all probes in the same direction, cannot be ruled out with certainty by determining parallelism. Therefore, we view the short-term follow-up (after 6 weeks) as the best measure of the success of ablation. In all 20 cases, technically successful complete ablation was accomplished by both CIRE and SIRE.

In our study of percutaneous microwave ablation of malignant liver tumors, we showed that robot support significantly reduced radiation exposure (Beyer et al., 2015). This applies to SIRE all the more as not only one, but several probes have to be inserted. Particularly great differences are seen in the DLP resulting from the use of CT fluoroscopy as well as in the total radiation exposure of the patient during the course of intervention (Table 4).

During SIRE, the time-consuming manual placement of the ablation electrodes is omitted. Instead, by using an aiming device in the specified insertion position, supported by 3D planning and patient co-registration, the ablation electrodes could be inserted *in situ* as one step.

In near future SIRE is probably not only a suitable method just for IRE of hepatic malignancies, but also for treatment of deep-seated tumors in other anatomical regions. For example one study has reported substantially prolonged survival for IRE as part of multimodal treatment of locally advanced pancreatic cancer (Martin et al., 2015). The placement of IRE electrodes in the pancreas is highly challenging because of the anatomical characteristics, especially the long access path and immediate proximity to large vessels which are respected by the IRE procedure. Therefore, we think that SIRE might be of high value for treatment of pancreatic cancer, especially because of the high accuracy and fast electrode placement, supported by strong 3D planning tools. Further studies should be conducted to evaluate the benefits of SIRE for different anatomical regions.

This study has some limitations. The single-center setup and the low number of procedures limits generalization of our results. In particular, reported results for the CIRE are very operator dependent and may vary accordingly in different centers.

## Conclusion

In summary, SIRE may be associated with a marked reduction of procedure length and high accuracy compared to CIRE. Stereotactic navigation has the potential to reduce radiation dose for the patient without increasing the risk of complications or impaired technical success compared to CIRE. Due to the high accuracy and focal nonthermal ablation mechanism, SIRE might have the potential to be translated into the treatment of deep-seated tumors in other anatomical regions, e.g., pancreatic cancer, in a near future.

##  Supplemental Information

10.7717/peerj.2277/supp-1 Data S1 Raw Data

10.7717/peerj.2277/supp-2 Supplemental Information 1 TREND Statement Checklist

## Additional Information and Declarations

### Data Availability

The following information was supplied regarding data availability: The raw data has been supplied as Supplemental Dataset.

### Clinical Trial Registration

The following information was supplied regarding Clinical Trial registration: ISRCTN55383115.
